# Acute Care Surgery Service Is Essential During a Nonsurgical Catastrophic Event, the COVID-19 Pandemic

**DOI:** 10.1177/0003134820972084

**Published:** 2020-12

**Authors:** Nikolay Bugaev, Horacio M. Hojman, Janis L. Breeze, Stanley A. Nasraway, Sandra S. Arabian, Sharon Holewinski, Benjamin P. Johnson

**Affiliations:** 1 Division of Trauma & Acute Care Surgery, 1867 Tufts University School of Medicine, Tufts Medical Center, Boston, MA, USA; 2 1867 Tufts Clinical and Translational Science Institute, Tufts University, and Institute for Clinical Research and Health Policy Studies, Tufts Medical Center, Boston, MA, USA; 3 Department of Surgery, 1867 Tufts University School of Medicine, Tufts Medical Center, Boston, MA, USA

**Keywords:** COVID-19 pandemic, acute care surgery service

## Abstract

**Background:**

The role of an acute care surgery (ACS) service during the COVID-19 pandemic is not well established.

**Methods:**

A retrospective review of the ACS service performance in an urban tertiary academic medical center. The study was performed between January and May 2020. The demographics, clinical characteristics, and outcomes of patients treated by the ACS service 2 months prior to the COVID surge (pre-COVID group) and during the first 2 months of the COVID-19 pandemic (surge group) were compared.

**Results:**

Trauma and emergency general surgery volumes decreased during the surge by 38% and 57%, respectively; but there was a 64% increase in critically ill patients. The proportion of patients in the Department of Surgery treated by the ACS service increased from 40% pre-COVID to 67% during the surge. The ACS service performed 32% and 57% of all surgical cases in the Department of Surgery during the pre-COVID and surge periods, respectively. The ACS service managed 23% of all critically ill patients in the institution during the surge. Critically ill patients with and without confirmed COVID-19 infection treated by ACS and non-ACS intensive care units during the surge did not differ in demographics, indicators of clinical severity, or hospital mortality:13.4% vs. 13.5% (*P* = .99) for all critically ill patients; and 13.9% vs. 27.4% (*P* = .12) for COVID-19 critically ill patients.

**Conclusion:**

Acute care surgery is an “essential” service during the COVID-19 pandemic, capable of managing critically ill nonsurgical patients while maintaining the provision of trauma and emergent surgical services.

**Level of Evidence:**

III.

**Study Type:**

Therapeutic.

## Background

The acute care surgery (ACS) is a relatively new surgical specialty that covers 3 clinical areas: trauma surgery, emergency general surgery (EGS), and surgical critical care (SCC). The main goals and organizational principles of the ACS model were initially outlined in the American Association for the Surgery of Trauma Ad Hoc Committee letter in 2005, which advocated for the creation of a new specialty that would provide comprehensive care to acutely ill surgical and trauma patients.^1^ Trauma and EGS patients treated at specialized trauma and newly established ACS centers have achieved superior clinical outcomes compared to patients treated in nonspecialized centers.^2-5^

The COVID-19 pandemic has challenged health care systems and hospitals with an increased demand for both human and material resources and has presented a unique opportunity to test the performance of the ACS model during this nonsurgical catastrophic event.^6^

During the COVID-19 surge in Boston from March to May 2020, the ACS service at an urban, tertiary, level I academic trauma center was assigned to continue coverage of all trauma, EGS, and SCC patients, with the added responsibility of taking care of critically ill nonsurgical patients including COVID-19 positive patients.

The aim of this study was to evaluate the utilization of our institution’s ACS service during the COVID-19 pandemic and to report the clinical outcomes of the critically ill COVID-19 patients treated by the ACS service during the COVID-19 surge. We hypothesize that the ACS model is applicable and effective in caring for both critically ill surgical and nonsurgical patients during a pandemic, like COVID-19, while still maintaining a provision of trauma and emergent surgical care.

## Methods

This study was approved by the Institutional Review Board of Tufts Medical Center (TMC); informed consent was waived. Tufts Medical Center is a 415-bed urban, tertiary academic medical center and an American College of Surgeons level I verified trauma center.

This study is a retrospective cohort analysis performed between January 25th and May 24th, 2020; it included all patients admitted to and managed by the ACS service at TMC during this time period. Among our cohort of patients, 2 study groups were defined. The pre-COVID control group consisted of all patients managed by the ACS service prior to the COVID-19 surge at our institution (January 25th to March 24th, 2020). The study group, termed as surge group, consisted of all patients, both surgical and nonsurgical, treated by the ACS service during the COVID-19 surge at our institution (March 25th to May 24th, 2020). March 25th was selected as the first COVID-19 positive patient was admitted to the surgical intensive care unit (SICU), managed by the ACS service, on this date.

Demographics and clinical characteristics of all trauma and EGS patients treated during these periods by the ACS service were collected from the hospital databases. The data regarding critically ill surgical patients were reported using MDN Phoenix database (Medical Decisions Network; Charlottesville, Virginia).^7^

### Staffing Model

The ACS service consists of 4 trauma surgeons who provide 24/7 in-hospital coverage for all trauma and EGS patients. The SICU is staffed by either one of the trauma surgeons not covering the ACS service or one of two intensivists (internal medicine and anesthesiology attendings). The attending responsible for the SICU performs daily morning rounds with the SICU multidisciplinary team and is then able to leave the hospital when the clinical situation allows. For the rest of the day, the SICU attending is not in-house, but is available for phone consultations and will return for in-person assessments when needed. The ACS surgical attending on call, who remains in-house, serves as a backup to the SICU attending and is able to evaluate all new admissions to the SICU and covers any emergency that requires immediate attention.

Changes in the staffing model were made during the COVID-19 surge in order to accommodate a greater influx of critically ill patients. The SICU was divided into 2 separate units: a COVID-19 SICU and an ACS/non-COVID ICU that accepted only confirmed COVID-19 negative patients. The anesthesiology attending who was a part of the regular SICU coverage in the pre-COVID period was reassigned to a newly organized COVID ICU. A chief surgical resident who had completed fellowship in and was board-eligible in critical care medicine was added to the team. This chief resident was always on call with 1 of the senior acute care surgeons, who was covering the ACS/non-COVID ICU and acted as a supervisor. Given the increasing number of critically ill COVID-19 patients with high acuity and clinical demands, the SICU attending coverage was changed from partial in-house presence to 24/7 in-house coverage for the COVID-19 SICU. The trauma surgeon assigned to the ACS service provided coverage of trauma and EGS cases, as well as the newly formed “clean” ACS/non-COVID ICU. The call schedule was then modified so that both teams, the COVID-19 SICU and the ACS /non-COVID ICU team, stayed in-house and worked in 12-hour shifts.

### Statistical Analysis

Statistical analysis was performed using Stata v16.1 (StataCorp, College Station, Texas). Categorical variables were described with frequencies and proportions, and continuous variables were described with means and standard deviations or medians and interquartile ranges depending on their distribution. Differences in demographic and clinical characteristics between the study groups were compared by either t-tests or Wilcoxon rank sum tests for continuous variables, as appropriate, and by χ^2^ or Fisher’s exact tests for categorical variables. All statistical testing was two-sided with α = .05 unless otherwise noted.

## Results

**Figure 1.**
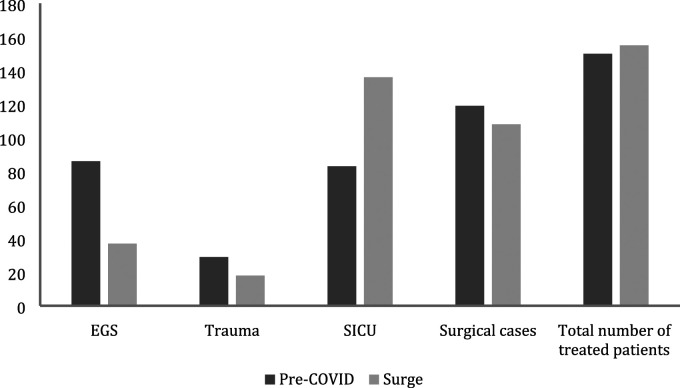
Patients treated by the acute care surgery service before and during COVID-19 surge. EGS, emergency general surgery; SICU, surgical intensive care unit.

The distribution of trauma, EGS, and critically ill patients managed by the ACS service during the study period is reported in Figure 1. Overall, the total number of patients managed by the ACS service increased by 3% during the surge period. Although the number of trauma and EGS patients decreased during this period, there was a dramatically higher number of critically ill patients (increase by 64%) treated by the ACS service during the surge period.

When the contribution to the total number of patients treated by the Department of Surgery was calculated, the proportion of patients treated by the ACS service increased from 40% pre-COVID to 67% during the surge period.

The overall number of surgical cases performed by the Department of Surgery during the surge period decreased by 50%; however, the number of surgical cases performed by the ACS service decreased by only 10%. The overall percentage of cases performed by the ACS service to all surgical cases performed by the Department of Surgery increased from 32% during pre-COVID to 57% during the surge. The 3 most common surgeries performed pre-COVID-19 were emergent laparotomy (35%), cholecystectomy (18%), and incision and drainage of wound/abscess (17%), whereas during the COVID-19 surge, this changed to tracheostomy and percutaneous endoscopic gastrostomy (PEG) (36%), incision and drainage of wound/abscess (28%), and emergent laparotomy (18%).

### Trauma/EGS Patients

The overall volume of trauma patients decreased during the surge period by 38% (Figure 1). There was a statistically significant decrease in age noted when comparing trauma population before and after the surge (mean age 48 ± 21 pre-COVID vs. 61 ± 21 during surge, *P* = .05). No other differences were found in terms of gender, injury severity score (median interquartile range) (17 (16-20) vs. 17 (9-18) *P* > .05), or injury patterns characterized by a frequency AIS > 2 between pre-COVID and surge times.

The EGS volume decreased by 57% during the surge period (Figure 1). No differences in age or gender were noted. The 3 most common admission diagnoses during pre-COVID-19 were complicated gallstone disease (21%), intestinal obstruction (20%), and acute appendicitis (15%). During the COVID surge, the 3 most common admission diagnoses were acute appendicitis (27%), complicated gallstone disease (22%), and intestinal perforation (16%). No COVID-19 infected patients were identified among the EGS patients treated.

### Comparisons of Critically Ill COVID-19 Patients Treated by ACS and Non-ACS Services.

During the COVID-19 surge, the total number of critically ill patients managed by the ACS service increased by 64% (Figure 1). The proportion of critically ill trauma (22.9% vs. 11.0%, *P* = .02) and EGS (21.7% vs. 6.6%, *P* < .001) patients treated by the ACS service was lower during the surge period. Overall, the ACS service managed 22.7% of all critically ill patients admitted to our institution during the surge period, and 27.8% of these critically ill patients had COVID-19 infection requiring mechanical ventilation (Table 1). No differences in age, gender, or race were identified between the pre-COVID group and surge group. The surge group had higher APACHE II. Overall, hospital mortality did not differ significantly between the pre-COVID and surge groups, 9.6% vs. 13.4%, *P* = .4.

**Table 1.** Critically Ill Patients Managed by the Acute Care Surgery Service.^a^

	Pre-COVID	Surge	*P*-value
	N = 83	N = 136	
Age, mean (SD)	60.3 (16.3)	59.0 (17.4)	.57
Male, n (%)	47 (56.6%)	79 (58.1%)	.83
Race/ethnicity, n (%)			.57
White	56 (67%)	92 (68%)	
Black	5 (6%)	16 (12%)	
Asian	9 (11%)	11 (8%)	
Hispanic	7 (8%)	11 (8%)	
Unknown/other	6 (7%)	6 (4%)	
APACHE II^a^, median (IQR)	15.0 (9.0-19.5)	19.5 (14.0-28.0)	.001
Acuity 2019 predicted mortality^b^, median (IQR)	6.0 (2.0-16.0)	13.0 (2.0-39.0)	.08
Patients on mechanical ventilation, n (%)	40 (48.2%)	61 (44.9%)	.63
Ventilator days^c^, median (IQR)	1.4 (.5-5.1)	3.2 (1.4-10.0)	.01
COVID-19 positive patients, n (%)	0 (.0%)	38 (27.9%)	<.001
ICU readmission, n (%)	6 (7.2%)	7 (5.1%)	.53
EGS patients, n (%)	18 (21.7%)	9 (6.6%)	<.001
Trauma patients, n (%)	19 (22.9%)	15 (11.0%)	.02
Patients from other non-COVID clinical services, n (%)	47 (56.6%)	112 (82.4%)	<.001
ICU LOS, median (IQR)	1.8 (1.0-4.1)	1.7 (.7-4.7)	.31
Hospital LOS, median (IQR)	7.5 (3.6-15.8)	6.8 (4.3-17.7)	.66
Disposition among those who survived to discharge			.63
Rehab	28 (37.3%)	44 (37.9%)	
AMA	1 (1.3%)	4 (3.4%)	
Home	44 (58.7%)	67 (57.8%)	
Hospice	2 (2.7%)	1 (.9%)	
Mortality	8 (9.6%)	18 (13.4%)	.40

Abbreviations: SD, standard deviation; IQR, interquartile range; APACHE II, Acute Physiology and Chronic Health Evaluation II score; EGS, emergency general surgery ; ICU, intensive care unit; LOS, length of stay; AMA, against medical advice.

a Acuity 2019 predicted mortality was calculated based on MDN Phoenix database (Medical Decisions Network; Charlottesville, Virginia).^7^

b Data were available for 60 patients in pre-COVID and 95 patients in the surge group.

c Data presented for those who were on mechanical ventilation.

When comparisons were made between critically ill patients treated by the ACS service and non-ACS ICU teams at our institution during the surge, no differences were noted in patient demographics, proportion of patients with COVID-19 infection, or total hospital mortality (13.4% vs. 13.5%, *P* = .99) (Table 2). Critically ill patients admitted to the ACS service, in comparison to non-ACS ICU services, had higher mean APACHE II score; however, no differences were found in the predicted mortality.

**Table 2.** Critically Ill Patients Managed by the ACS ICU and non-ACS ICUs During COVID Surge.^a^

	ACS ICU	Non-ACS ICUs	*P*-value
	N = 136	N = 463	
Age, mean (SD)	59.0 (17.4)	61.3 (16.8)	.17
Male, n (%)	79 (58.1%)	285 (61.6%)	.47
Race/ethnicity, n (%)			.85
White	92 (68%)	294 (63%)	
Black	16 (12%)	66 (14%)	
Asian	11 (8%)	48 (10%)	
Hispanic	11 (8%)	35 (8%)	
Unknown/other	6 (4%)	20 (4%)	
APACHE II^a^, median (IQR)	19.5 (14.0-28.0)	16.0 (11.0-23.0)	.01
Acuity 2019 predicted mortality^b^, median (IQR)	13.0 (2.0-39.0)	9.0 (3.0-31.0)	.71
Patients on mechanical ventilation, n (%)	61 (44.9%)	180 (38.9%)	.21
Ventilator days^c^, median (IQR)	3.2 (1.4-10.0)	5.7 (2.4-11.5)	.03
COVID-19 positive patients, n (%)	38 (27.9%)	138 (29.8%)	.67
ICU readmission, n (%)	7 (5.1%)	17 (3.7%)	.44
ICU LOS, median (IQR)	1.7 (.7-4.7)	2.7 (1.1-6.7)	<.001
Hospital LOS, median (IQR)	6.8 (4.3-17.7)	7.0 (3.6-16.8)	.68
Disposition among those who survived to discharge			.08
Rehab	44 (37.9%)	111 (28.3%)	
AMA	4 (3.4%)	6 (1.5%)	
Home	67 (57.8%)	265 (67.6%)	
Hospice	1 (.9%)	10 (2.6%)	
Mortality	18 (13.4%)	61 (13.5%)	.99

Abbreviations: ACS, acute care surgery; ICU, intensive care unit; SD, standard deviation; IQR, interquartile range; APACHE II, Acute Physiology and Chronic Health Evaluation II score; LOS, length of stay; AMA, against medical advice.

a Acuity 2019 predicted mortality was calculated based on MDN Phoenix database (Medical Decisions Network; Charlottesville, Virginia).^7^

b Data were available for 95 patients in ACS ICU group and 185 patients in non-ACS ICUs group.

c Data presented for those who were on mechanical ventilation.

The analysis of critically ill patients who had documented COVID-19 infection demonstrated similar results in terms of demographics, severity of critical illness, predicted mortality, and total mortality (13.9% vs. 27.4%, *P* = .12) between those treated by the ACS service and those treated by non-ACS ICU services (Table 3).

**Table 3.** Critically Ill COVID-19 Positive Patients Managed by the ACS ICU and non-ACS ICUs.^a^

	ACS ICU	Non-ACS ICUs	*P*-value
	N = 36	N = 64	
Age, mean (SD)	58.1 (18.8)	59.0 (15.7)	.80
Male, n (%)	19 (52.8%)	41 (64.1%)	.27
Race/ethnicity, n (%)			.88
White	18 (50%)	28 (44%)	
Black	8 (22%)	12 (19%)	
Asian	4 (11%)	12 (19%)	
Hispanic	5 (14%)	9 (14%)	
Unknown/other	1 (3%)	3 (6%)	
APACHE II, median (IQR)	26.0 (17.5-29.0)	20.5 (11.5-27.0)	.04
Acuity 2019 predicted mortality, median (IQR)	22.0 (9.0-58.5)	22.0 (4.5-50.0)	.32
Patients on mechanical ventilation, n (%)	27 (75.0%)	43 (67.2%)	.41
Ventilator days^b^, median (IQR)	7.7 (3.2-16.0)	11.6 (3.7-17.5)	.32
ICU readmission, n (%)	3 (8.3%)	2 (3.1%)	.25
ICU LOS, median (IQR)	6.6 (1.9-15.7)	6.0 (1.6-19.6)	.78
Hospital LOS, median (IQR)	21.6 (7.8-40.7)	11.8 (7.0-29.9)	.08
Disposition among those who survived to discharge			.02
Rehab	23 (63.9%)	20 (32.3%)	
Home	8 (22.2%)	24 (38.7%)	
Hospice	0 (.0%)	1 (1.6%)	
Mortality	5 (13.9%)	17 (27.4%)	.12

Abbreviations: ACS, acute care surgery; ICU, intensive care unit; SD, standard deviation; IQR, interquartile range; APACHE II, Acute Physiology and Chronic Health Evaluation II score; LOS, length of stay.

a Acuity 2019 predicted mortality was calculated based on MDN Phoenix database (Medical Decisions Network; Charlottesville, Virginia).^7^

b Data presented for those who were on mechanical ventilation.

## Discussion

This study presents the results of a successful implementation of the ACS model in an urban, tertiary academic level 1 trauma center during a nonsurgical catastrophic event, the COVID-19 pandemic. Although the volume of EGS and trauma patients decreased during the COVID-19 surge, the ACS providers successfully adapted to managing the influx of critically ill nonsurgical COVID-19 patients while still maintaining the surgical productivity of the ACS service. Clinical outcomes of the critically ill patients treated by ACS service during COVID-19 surge did not significantly differ from other nonsurgeon-managed ICUs at our institution.

The ACS model was initially introduced to address a nationwide shortage in the coverage of acutely ill surgical patients. The main goal of this model was to provide improved continuity of care to trauma, EGS, and critically ill surgical patients. Acute care surgeons are required to have a wide spectrum of clinical skills, allowing them to provide a high quality of both surgical and intensive medicine care to a diverse population of patients.^1^ By its nature, the ACS model is predominantly designed to be utilized in emergent surgical settings that carry a great deal of clinical unpredictability and complexity. It is also designed to be performed as a shift-type work schedule that requires high-quality handoff between providers and an ability to adjust to a fast-changing clinical situation.^2^ During the COVID-19 surge, which occurred at our institution from April to May 2020, we saw a significant increase in the number of critically ill nonsurgical patients admitted. A shortage of nonsurgical ICU beds and medical intensive care staff led to the utilization of the surgical ICU in the management of critically ill nonsurgical patients. With some adjustments in the ACS call schedule, we managed to accommodate the influx of critically ill nonsurgical patients while maintaining surgical coverage for trauma and EGS patients. As opposed to many other centers, our 4 trauma surgeons remained dedicated to all 3 tenants of ACS: EGS, trauma surgery, and critical care rather than being allocated to only caring for 1 of those clinical areas.^2^ In addition, given the overwhelmingly higher number of COVID-19 patients and a need to relocate both human and material resources, we temporarily changed trauma activations criteria allowing ED physicians to have a greater role managing lower acuity trauma cases without routine trauma team activation.

During the COVID-19 surge, we observed the previously described phenomenon where a decrease in surgical volume is compensated by an increase in the number of ICU patients.^6,8^ Uniquely approaching the recent COVID-19 surge as a nonsurgical “mass casualty event” helped us to reallocate resources to clinical “hot spots” while continuing to provide adequate coverage across all patient populations managed by the ACS service.

The surge period was characterized by a higher overall proportion of critically ill patients admitted to our institution. The majority of patients admitted to the surgical ICU during the surge were not surgical patients. The admission of nonsurgical critically ill patients to ICU units was dictated solely by bed availability and not by particular ICU specialty, with the exception that all critically ill surgical patients were exclusively admitted to the SICU and managed by the ACS service. The increased number of the critically ill patients during the COVID-19 surge led to the higher acuity of critically ill patients admitted to the ACS service during that period in comparison to the pre-COVID time. Ultimately, there was no statistically significant difference in mortality among critically ill ACS patients before and during the COVID-19 surge (13.6% vs. 9.4%, *P* > .05). When comparing patients treated in ACS- and non-ACS-managed ICUs during the surge period, there were no significant differences noted in the distribution of patients in terms of critical illness indicators or overall mortality. However, among critically ill COVID-19 positive patients treated by the ACS service, there was a nonsignificant trend toward lower mortality (13.9 vs. 27.4%, *P* = .12). The outcomes of this specific group of patients were in line with previously reported outcomes in critically ill COVID-19 patients from other institutions.^9-11^

At our institution, no elective surgeries were allowed during the COVID surge, except for those patients in whom a surgery delay would lead to a permanent health damage.^12^ As a result, we noted that more than 50% of all surgical cases in the Department of Surgery during the COVID surge were performed by the ACS service. The urgent and emergent nature of surgical cases typically performed by the ACS service resulted in a disproportionately high surgical contribution of the ACS service during the COVID-19 surge. The frequency of different types of surgical cases performed also shifted during this period. The increase in the number of patients on prolong mechanical ventilation during the COVID-19 surge led to a raising need for tracheostomies and PEG tubes in those patients. This resulted in these 2 procedures being the most common surgery performed during the COVID-19 surge, as opposed to exploratory laparotomy in the pre-COVID period. The ACS service was the leading provider of tracheostomy and PEG tube placement at our institution.

Our study has few limitations. The retrospective nature of this study did not allow us to perform a detailed analysis of the included patients and the treatment decisions that were made. One of the main limitations we encountered was a significant amount of missing data in terms of APACHE II and predicted mortality. The results of the study were not possible to adjust to frequently changing COVID-19 prevention and treatment protocols.

## Conclusion

The ACS model can be successfully implemented during a nonsurgical catastrophic event, as demonstrated at our institution during the COVID-19 pandemic. Acute care surgery is an “essential” surgical service capable of managing critically ill nonsurgical patients while maintaining provision of trauma and emergent surgical services.
